# RNA-Based Assay for Next-Generation Sequencing of Clinically Relevant Gene Fusions in Non-Small Cell Lung Cancer

**DOI:** 10.3390/cancers13010139

**Published:** 2021-01-04

**Authors:** Caterina De Luca, Francesco Pepe, Antonino Iaccarino, Pasquale Pisapia, Luisella Righi, Angela Listì, Lorenza Greco, Gianluca Gragnano, Severo Campione, Gianfranco De Dominicis, Fabio Pagni, Roberta Sgariglia, Mariantonia Nacchio, Rossella Tufano, Floriana Conticelli, Elena Vigliar, Claudio Bellevicine, Diego Luigi Cortinovis, Silvia Novello, Miguel Angel Molina-Vila, Rafael Rosell, Giancarlo Troncone, Umberto Malapelle

**Affiliations:** 1 Department of Public Health, University of Naples Federico II, 80131 Naples, Italy; caterina.deluca@unina.it (C.D.L.); francesco.pepe4@unina.it (F.P.); antiaccc@hotmail.com (A.I.); pasquale.pisapia@unina.it (P.P.); lorenza.greco6@gmail.com (L.G.); gianluca.gragnano@unina.it (G.G.); roberta.sgariglia@unina.it (R.S.); mariantonia.nacchio@unina.it (M.N.); floriana.conticelli@unina.it (F.C.); elena.vigliar@unina.it (E.V.); claudio.bellevicine@unina.it (C.B.); umberto.malapelle@unina.it (U.M.); 2 Department of Oncology, San Luigi University Hospital, University of Turin, 10043 Orbassano, Italy; luisella.righi@unito.it (L.R.); alisti@live.it (A.L.); 3 Anatomic Pathology, A.O.R.N. Antonio Cardarelli, 80131 Naples, Italy; severo.campione@aocardarelli.it (S.C.); gianfranco.dedominicis@aocardarelli.it (G.D.D.); silvia.novello@unito.it (S.N.); 4 Department of Medicine and Surgery, San Gerardo Hospital, University of Milano-Bicocca, 20900 Monza, Italy; fabio.pagni@unimib.it (F.P.); d.cortinovis@asst.monza.it (D.L.C.); 5 CEINGE Biotecnologie Avanzate, Via Gaetano Salvatore 486, 80131 Naples, Italy; rossella.tufano@libero.it; 6 Laboratory of Oncology, Pangaea Oncology, 08028 Barcelona, Spain; mamolina@panoncology.com; 7 Cancer Biology and Precision Medicine Program Catalan Institute of Oncology, Germans Trias i Pujol Health Sciences Institute and Hospital Badalona, 08916 Barcelona, Spain; rrosell@iconcologia.net

**Keywords:** predictive molecular pathology, next generation sequencing, gene fusions, targeted therapy, NSCLC

## Abstract

**Simple Summary:**

Gene fusions represent novel predictive biomarkers for advanced Non Small Cell Lung Cancer (NSCLC) patients. In this study, we developed and validated a narrow Next Generation Sequencing gene panel able to cover *ALK, ROS1, RET* and *NTRK* gene fusions and *MET* splicing events in advanced-stage NSCLC patients. Overall, our RNA fusion panel was able to detect all fusions and a splicing event harbored in a RNA pool diluted up to 2 ng/µL. In addition, It also successfully analyzed 46 (95.8%) out of 48 routine samples previously characterized by conventional non - NGS technology, representing a robust tool for routine setting.

**Abstract:**

Gene fusions represent novel predictive biomarkers for advanced non-small cell lung cancer (NSCLC). In this study, we validated a narrow NGS gene panel able to cover therapeutically-relevant gene fusions and splicing events in advanced-stage NSCLC patients. To this aim, we first assessed minimal complementary DNA (cDNA) input and the limit of detection (LoD) in different cell lines. Then, to evaluate the feasibility of applying our panel to routine clinical samples, we retrospectively selected archived lung adenocarcinoma histological and cytological (cell blocks) samples. Overall, our SiRe RNA fusion panel was able to detect all fusions and a splicing event harbored in a RNA pool diluted up to 2 ng/µL. It also successfully analyzed 46 (95.8%) out of 48 samples. Among these, 43 (93.5%) out of 46 samples reproduced the same results as those obtained with conventional techniques. Intriguingly, the three discordant results were confirmed by a CE-IVD automated real-time polymerase chain reaction (RT-PCR) analysis (Easy PGX platform, Diatech Pharmacogenetics, Jesi, Italy). Based on these findings, we conclude that our new SiRe RNA fusion panel is a valid and robust tool for the detection of clinically relevant gene fusions and splicing events in advanced NSCLC.

## 1. Introduction

Non-small cell lung cancer (NSCLC) represents the leading cause of cancer mortality worldwide [1]. Unfortunately, being commonly diagnosed in the advanced stages of the disease, it is very difficult to treat. Over the last decade, an upturn in NSCLC treatment has been the identification and use of several biomarkers to predict patients’ response to personalized treatments. Indeed, biomarker-based personalized treatments have significantly improved progression free survival (PFS) in advanced stage NSCLC patients harboring several actionable point mutations, indels, and gene rearrangements [2,3,4,5,6,7,8]. One such biomarker is Anaplastic Lymphoma Kinase (*ALK*) rearrangement (3–6%), which can predict marked sensitivity to crizotinib, alectinib, and brigatinib treatments [5,9,10,11,12,13,14]. Another important biomarker is ROS Proto-Oncogene 1 Receptor Tyrosine Kinase (*ROS1*) gene fusion (1–2%), which is known to respond remarkably well to crizotinib [15,16,17]. Similarly, Neurotrophic Receptor Tyrosine Kinase (*NTRK*) fusions (<1%) respond to entrectinib [18,19,20,21]. Lastly, *REarranged during Transfection* (*RET*) gene fusions (1–2%) [22,23] respond well to selpercatinib and pralsetinib [24,25].

However, detecting therapeutically relevant gene fusions may be time-consuming and costly, and, in some specific cases, may require high-quality histological samples. For instance, fluorescence in situ hybridization (FISH) and immunohistochemistry (IHC) require a series of consecutive assays to test for all these gene fusions, and, in the case of *RET* fusion samples, IHC does not yield accurate results. 

Conversely, next generation sequencing (NGS) comprehensively and simultaneously evaluates all actionable targets in a single run. [26] Furthermore, besides specifying fusion variants, it can also detect de novo gene fusions and other RNA alterations, including mutations in the MET Proto-Oncogene, Receptor Tyrosine Kinase (*MET*) exon 14 skipping isoform [27,28,29]. However, one drawback of the “wide” NGS gene panels for NSCLC is that they comprise not only vast numbers of actionable standard of care targets but also extrapulmonary and investigational DNA and/or RNA biomarkers, thus rendering these panels rather impracticable in routine clinical practice. Conversely, “narrow” gene panels, [30] which cover only NSCLC targets make NGS more cost-effective and much easier to implement in routine practice. Not surprisingly, there is a much higher request for narrow targeted panels than for larger ones [3]. 

In our routine clinical practice, we have recently adopted a “narrow” DNA custom gene panel to sequence several *somatic* mutations, in particular Epidermal Growth Factor Receptor (*EGFR*), Kirsten Rat Sarcoma Viral Oncogene Homolog (*KRAS*), Neuroblastoma RAS Viral Oncogene Homolog (*NRAS*), and V-Raf Murine Sarcoma Viral Oncogene Homolog B (*BRAF*) [31]. Moreover, considering that gene fusions and splicing events are equally important cancer driver mutations in NSCLC, we sought to improve our diagnostic performance by adopting the same approach to design a “narrow” RNA custom gene panel, which we called SiRe fusion, specially designed to detect the most clinically relevant gene fusion and splicing events in advanced-stage lung cancer. Thus, the aim of the present study was to validate our novel “narrow” RNA custom gene panel for NGS analysis of *ALK*, *ROS1*, *NTRK*, and *RET* rearrangements, as well as the *MET* exon 14 skipping mutation. 

## 2. Materials and Methods

### 2.1. Panel Design and Library Preparation Protocol

To cover *ALK*, *ROS1*, *RET*, and *NTRK* gene fusions, as well as and *MET* exon 14 skipping alterations simultaneously, we developed a fusion primer pool by using AmpliSeq designer software v.7.4 which uses hg19 as a reference sequence. The panel, named SiRe fusion, was designed to sequence cDNA obtained after retro-transcription of RNA extracted from formalin fixed and paraffin embedded (FFPE) sections, cytopathological specimens, and body fluid samples of NSCLC patients. Several experimental and analytical steps were carried out to assess the reference range, the minimal input of cDNA, the limit of detection (LoD), and the overall practicability of this panel in routine practice. 

Libraries were constructed and purified on the Ion Chef Instrument (ThermoFisher Scientifics, Waltham, MA, USA) according to the manufacturer’s instructions. In brief, 6 µL of cDNA (with an optimal concentration of 2 ng/microliter) was dispensed on the Ion Code plates and amplified with Ion AmpliSeq DL8 (ThermoFisher Scientifics). cDNA was amplified with 24 cycles, whereas the library was re-amplified with 7 PCR cycles after barcoding, under the thermal conditions recommended by the manufacturer. Purified libraries from RNA samples were diluted to 60 pM and pooled. They were then re-loaded onto the Ion Chef Instrument, and the templates were prepared with the S5 510–520–530 Kit-Chef (ThermoFisher Scientifics). Finally, templates were loaded onto the 520 chip and sequenced on the S5 NGS platform (ThermoFisher Scientifics). The data were interpreted with a proprietary pipeline, developed by the Department of Public Health, University of Naples Federico II, and visualized on Ion Reporter Software (ThermoFisher Scientifics). The bioinformatic analysis was carried out by using Ion Reporter Ion Reporter™ 5.16.0.2 with customized version of AmpliSeq RNA Lung Fusion—w1.2—Single Sample. All the panel design files were reported in the Supplementary Material (Supplementary Files S1–S9). Any additional information on the panel design and data analysis is available on request.

### 2.2. Molecular Reference Standards

The custom RNA fusion gene panel was validated as follows. Minimal cDNA input and LoD were assesses by employing, different molecular reference standards. Ethanol fixed slides prepared from cells simultaneously harboring Echinoderm Microtubule-Associated Protein-Llike 4 [*EML4*](10)-*ALK*(20), Solute Carrier Family 34 Member 2 [*SLC34A2*](4)-*ROS1*(32), Coiled-Coil Domain Containing 6 [*CCDC6*](1)-*RET*(12) and Tropomyosin 3 [*TPM3*](8)-*NTRK1*(10)] were purchased from Horizon Diagnostics (HDx Cambridge, United Kingdom). In addition, several cell lines, each carrying a specific gene fusion, were purchased from the American Type Culture Collection. RNA was extracted from of the following cell lines: H3122 [*EML4*(13)-*ALK*(20)], H2228 [*EML4*(6)-*ALK*(20)], Hs746T (*MET* exon 14 skipping), H596 (*MET* exon 14 skipping), HCC-78, [*SLC34A2*(4)-*ROS1*(32)]; LC2ad [*CCDC6*(1)-*RET*(12)], EBC-1, (*MET* Amplification), SUDHL-1, (Nucleophosmin 1 [*NPM1*]-*ALK***,** and NTRK1cl [*TPM3*(8)-*NTRK1*(10)] (Supplementary Table S1). Finally, the extracted RNA was used to prepare reference standards at different dilutions (Supplementary Table S2).

### 2.3. RNA Extraction, Retrotrascription and Quantification

For molecular reference standards, RNA was extracted with the All Prep DNA- RNA Mini Kit (Qiagen, Hilden, Germany), according to the manufacturer’s instructions, and re-suspended in 50 µL of RNAsi/DNAsi free water (Ambion, ThermoFisher Scientifics). RNA was directly extracted from the above mentioned cell lines (Pangaea Biotech, Barcelona, Spain) with the High Pure RNA Isolation kit (Roche Diagnostics, Basel, Switzerland) according to the manufacturer’s instructions. TapeStation 4200 (Agilent Technologies, Santa Clara, CA, USA), a microfluidic based technology, evaluated the RNA concentration (ng/µL) and RNA integrity number (RIN). The RNA was eluted in 30 μL of RNAsi/DNAsi free water (Ambion, ThermoFisher Scientifics). Retro-transcription was carried out with SuperScript IV VILO Master Mix (ThermoFisher Scientifics) according to the manufacturer’s instructions. The cDNA was then quantified by using the D1000 Genomic assay on TapeStation 4200 (Agilent Technologies) (Supplementary Figures S1 and S2). Our custom panel NGS analysis was performed as above described; the results were further confirmed by an automated real time polymerase chain reaction (RT-PCR) approach (Easy PGX platform, Diatech Pharmacogenetics, Jesi, Italy) for *ALK*, *ROS1*, *RET,* and *NTRK* gene fusions, as well as the *MET* exon 14 skipping mutation.

### 2.4. Validation of the SiRe Fusion Panel in Routine Samples

**Figure 1:**
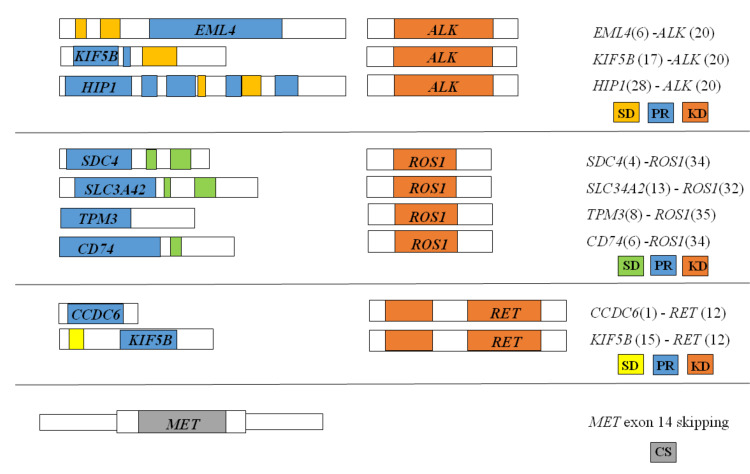
Schematic representation of gene fusions reported in a routine sample set: The kinase domain is in orange; the promoter region is in blue; the structural domains are in yellow, ochre, and green; the coding sequence is in grey. Abbreviations: *ALK*: Anaplastic Lymphoma Kinase; *CCDC6*: Coiled-Coil Domain Containing 6; *CD74*: CD74 Molecule, Major Histocompatibility Complex, Class II Invariant Chain; CS: sequence; *EML4*: Echinoderm Microtubule Associated Protein Like 4; *HIP1*: Huntingtin Interacting Protein 1; KD: kinase domain; *KIF5B*: Kinesin Family Member 5B; *MET*: MET Proto-Oncogene, Receptor Tyrosine Kinase; PR: promoter region; *RET*: Rearranged During Transfection; *ROS1*: ROS Proto-Oncogene 1, Receptor Tyrosine Kinase; SD: structural domain; *SLC34A2*: Solute Carrier Family 34 Member 2; *SDC4*: Syndecan 4; *TPM3*: Tropomyosin 3.

**Figure 2:**
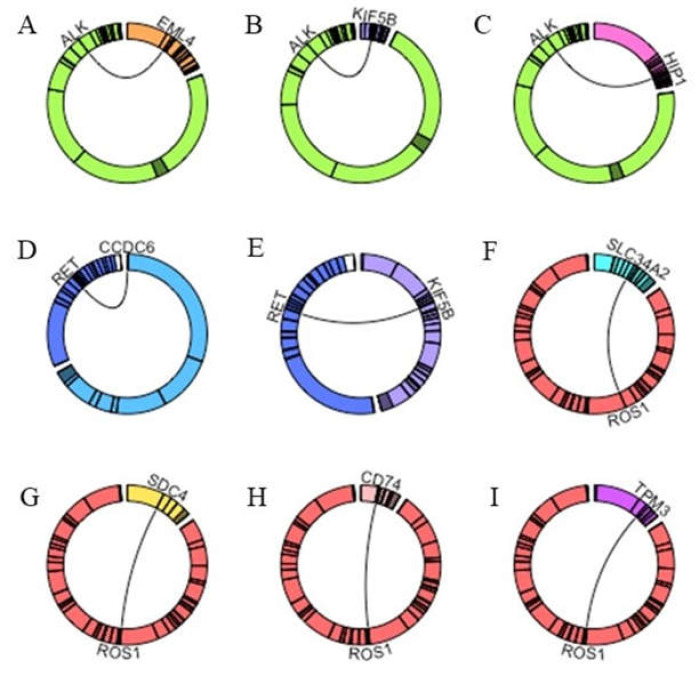
Circos plots representation of the gene fusions reported in routine sample set by using the R circlize package (R Core Team v3.6.1) [32]. (**A**–**C**) shows *ALK* translocations and corresponding fusion partners detected in analyzed samples. (**D**,**E**) shows *RET* fusions and gene partners identified in sample population. (**F**–**I**) shows *ROS1* translocations and fusion partners inspected in analyzed samples. Abbreviations: *ALK*: Anaplastic Lymphoma Kinase; *CCDC6*: Coiled-Coil Domain Containing 6; *CD74*: CD74 Molecule, Major Histocompatibility Complex, Class II Invariant Chain; CS: sequence; *EML4*: Echinoderm Microtubule Associated Protein Like 4; *HIP1*: Huntingtin Interacting Protein 1; KD: kinase domain; *KIF5B*: Kinesin Family Member 5B; *MET*: MET Proto-Oncogene, Receptor Tyrosine Kinase; PR: promoter region; *RET*: Rearranged During Transfection; *ROS1*: ROS Proto-Oncogene 1, Receptor Tyrosine Kinase; SD: structural domain; *SLC34A2*: Solute Carrier Family 34 Member 2; *SDC4*: Syndecan 4; *TPM3*: Tropomyosin.

To evaluate the performance of our SiRe fusion panel in a clinical setting, we retrospectively selected archived lung adenocarcinoma histological and cytological (cell blocks) samples harboring *ALK*, *ROS1*, *RET*, and *NTRK* gene fusions, as well as the *MET* exon 14 skipping mutation. This retrospective analysis was based on previous diagnoses of gene rearrangements involving *ALK*, *ROS1*, and *RET* made by various Italian institutions by immunohistochemistry (IHC), fluorescent in situ hybridization (FISH), and NGS analyses (University of Naples Federico II, Naples, Italy; A.O.R.N. Antonio Cardarelli, Naples, Italy; San Luigi University Hospital, University of Turin, Orbassano, Italy; San Gerardo Hospital, University of Milano-Bicocca, Monza, Italy) S. Sample characteristics and gene alterations are reported in both Table 1 and Figure 1 and Figure 2.

## 3. Results

### 3.1. Panel Design

The fusion primer pool for SiRe was designed with the AmpliSeq designer software v.7.4.1., which uses hg19 as a reference sequence. Overall, 16 samples in 520 chips on a S5/S5XL Ion Torrent platform (ThermoFisher Scientifics), were run simultaneously. Remarkably, the pool of 91 primer pairs covered all the targeted gene fusions, reaching a 5000× coverage for each amplicon. Moreover, since the fusion primer pool was fully compatible with the custom NSCLC DNA primer pool, a DNA/RNA pool was generated in a single NGS run. The DNA libraries can be processed in parallel with RNA libraries, under the same thermal and sequencing conditions. The RNA primer pool, as reported in the method section, was developed to be fully integrable with SiRe primers pool [31]. The SiRe results are reported in Supplementary Tables S1 and S2 and Supplementary File S3. 

### 3.2. Reference Range and Fixation Modalities

All samples processed for the feasibility test passed the quality filters. The NGS run parameters ensured an optimal analytical performance of the RNA fusion panel, as evidenced by its ability to reliably detect clinically relevant rearrangements in *ALK*, *ROS1*, *RET*, and *NTRK*, as well as *MET* exon 14 skipping mutations. Taking into account all the mutant cases, we obtained, on average, 99.70% (ranging from 99.13% to 99.98%) on-target reads, 103.55 bp read lengths (ranging from 93.00 to 114.00), 311,798.44 mapped reads (ranging from 60,530.00 to 948,223.00), and 318415.89 coverage uniformity (ranging from 60,562.00 to 96,378.00). The SiRe RNA fusion panel was able to detect *ALK*, *ROS1*, *RET*, and *NTRK* rearrangements, as well as *MET* exon 14 skipping mutations. All the results, including the fusion partner genes, are reported in Supplementary Tables S3–S7. 

Adequate quality and quantity RNA and cDNA input were extracted from methanol and ethanol fixed cell lines. Moreover, NGS analysis correctly detected all engineered mutations in both groups (Supplementary Tables S3 and S4) and no false positive results were observed in the wild type groups (Supplementary Tables S5 and S6). The Easy PGX platform further confirmed all the mutations on residual RNA extracted from fusion-positive *ALK*, *ROS1*, *RET* samples, as well as the *MET* exon 14 skipping kit and the *NTRK* fusion kit (Supplementary Table S7).

The LoD was determined by using RNA extracted from four cell lines, which were diluted to obtain 50%, 25%, and 10% of translocated *EML4*-*ALK* v3 and *SLC34A2*-*ROS1*, as reported in the Materials and Methods section. In all instances, the assay was able to detect both *ALK* and *ROS1* fusions. 

### 3.3. Optimization of Cell Line Quantity and Limit of Detection

The RNA isolated from all the cell lines (Supplementary Table S2) was pooled to generate five different RNA input quantity points (20 ng/µL, 10 ng/µL, 2 ng/µL, 0.5 ng/µL, 0.1 ng/µL), and analyzed in the aforementioned manner. Overall, we obtained, on average, 73.97% (ranging from 64.51% to 85.60%) on-target reads, 93.00 bp (ranging from 48.00 to 117.00) read lengths, 21427.80 (ranging from 13,642.00 to 27,230.00) mapped reads, and 20432.40 (ranging from 28,229.00 to 13,784.00) coverage uniformity. The SiRe RNA fusion panel was able to detect all fusions and the splicing event harbored by the RNA pool diluted up to 2 ng/µL. (Supplementary Table S2).

### 3.4. SiRe Fusion Panel in a Routine Setting and Evaluation of Discordant Samples 

The applicability of our SiRe fusion panel to a routine clinical setting was assessed by testing detection performance in 19 cell blocks and 29 histological (n = 15 resection and n = 14 biopsies) fusion positive samples of advanced stage NSCLC patients (25 men and 23 women; mean age: 56.0 years) retrieved from the archives. Sample characteristics, mutational status, and technologies are reported in Table 1. Overall, our SiRe fusion panel successfully analyzed 46 (95.8%) out 48 samples. Among these, 43 (93.5%) out of 46 samples accurately reproduced the results obtained with conventional techniques. Only three (6.5%) out of 46 instances yielded discordant results. These discordant results were further confirmed by CE-IVD automated RT-PCR analysis (Easy PGX platform, Diatech Pharmacogenetics) for *ALK*, *ROS1*, *RET,* and *NTRK* fusions, as well as *MET* exon 14 skipping mutations. The specific characteristics of the fusions and relative read counts are reported in Table 1. 

## 4. Discussion

Advanced lung cancer, like many other malignancies, is driven by various genetic changes, including gene fusions and splicing events. Thus, the identification of therapeutically relevant gene fusions (*ALK*, *ROS1*, *RET* and *NTRK*) and *MET* exon 14 skipping mutations is pivotal in choosing correct management of advanced-stage NSCLC [3,6]. Currently, IHC and FISH represent the gold standard methodologies for identifying these alterations, as evidenced in different clinical trials [5,26]. However, in a substantial number of cases FISH and IHC yield discordant results [33]. In this setting, RNA-sequencing plays a key role in defining the molecular assessment of tumor samples and in making treatment decisions for patients affected by advanced NSCLC. In addition, the implementation of NGS in routine clinical practice is key to making do with the scant amount of analyzable tissue from these patients, when one considers the increasing number of clinically relevant biomarkers tested in clinical practice. Indeed, NGS enables, not only to test DNA based alterations, but also to evaluate relevant gene fusions simultaneously. Today, it is no longer sustainable, especially at the local level, to test clinically relevant biomarkers for NSCLC patients by using IHC-based algorithms or FISH. More recently, the European Society for Medical Oncology (ESMO) recommends the adoption of NGS technology in patient with advanced non-squamous NSCLC to analyze not only DNA-based biomarkers but also RNA-based gene rearrangements and other alterations, such as *MET* exon 14 skipping mutations. To this end, an integrated RNA- and DNA-based NGS approach should be preferred [34]. 

In our experience, we demonstrated that our narrow SiRe NGS fusion panel is a reliable tool to detect clinically relevant gene fusions and *MET* exon 14 skipping alterations in routine samples of lung cancer. Remarkably, our new custom panel accurately reproduced (93.5%) the results obtained with conventional techniques. Considering the three discrepant cases between NGS and FISH analysis, it should be considered that in all instances a “borderline” FISH positive results was obtained, featuring a percentage of rearranged nuclei around 15%. In addition, our approach was able to identify all gene fusions and the splicing event harbored in the RNA pool extracted from cell lines, even when the RNA concentration was as low as 2 ng/µL. Taking into account this parameter, it is conceivable that the inadequate NGS results may be related to low quality RNA. This underlines its applicability to cytological samples and small histological biopsies, which represent 80% of all advanced stage NSCLC sample types.

In conclusion, the study fully validates the feasibility of integrating a DNA (SiRe^®^) and RNA (SiRe fusion) primer pool in an Ampliseq based approach to cover all the clinically relevant NSCLC alterations (E*GFR*, *KRAS*, *BRAF*, *ALK*, *ROS1*, *RET*, *NTRK*, and *MET*ex14) simultaneously in a single run, regardless of the small sample size of available tissue. A further study is ongoing to evaluate in addition to the specificity also the sensitivity of our panel on a wide cohort of patients, including not only mutated but also wild type cases. 

## Figures and Tables

**Table 1:** Patients’ characteristics and *SiRe* results.

	Patients and Sample Characteristics					Fusions Detected with Conventional Methods		Fusions Detected with SiRe		
N	Sex	Age	Sample Type	Sample Sub-Type	% Neoplastic Cells	Fused Gene	Detection Method	Locus	Genes (Exon)	Reads Count
1	F	60	Histological	Resection	80%	*ALK*	IHC	chr2:42491871-chr2:29446394	*EML4*(6)-*ALK*(20)	5321
2	M	62	Histological	Resection	60%	*ALK*	IHC	chr2:42492091-chr2:29446394	*EML4*(6)-*ALK*(20)	6285
3	M	47	Histological	Resection	70%	*ALK*	IHC	chr2:42491871-chr2:29446394	*EML4*(6)-*ALK*(20)	1662
4	M	76	Histological	Biopsy	30%	*ALK*	IHC	chr2:42522656-chr2:29446394	*EML4*(13)-*ALK*(20)	2391
5	F	44	Histological	Resection	60%	*ALK*	IHC	chr20:43959006-chr6:117645578	*SDC4*(4)-*ROS1*(34)	94
6	F	63	Histological	Resection	70%	*ROS1*	IHC	chr5:149784243-chr6:117645578	*CD74*(6)-*ROS1*(34)	28,678
7	M	59	Histological	Biopsy	60%	*ALK*	IHC	chr2:42552694-chr2:29446394	*EML4*(20)-*ALK*(20)	4353
8	M	69	Histological	Biopsy	40%	*ALK*	IHC	chr10:32311068-chr2:29446394	*KIF5B*(17)-*ALK*(20)	9641
9	F	48	Histological	Resection	80%	*ROS1*	IHC	chr5:149784243-chr6:117645578	*CD74*(6)-*ROS1*(34)	4347
10	M	45	Histological	Biopsy	40%	*ALK*	IHC	chr2:42522656-chr2:29446394	*EML4*(13)-*ALK*(20)	5821
11	M	36	Histological	Resection	80%	*ALK*	IHC	chr2:42522656-chr2:29446394	*EML4*(13)-*ALK*(20)	2279
12	M	40	Histological	Biopsy	40%	*ALK*	IHC	chr2:42522656-chr2:29446394	*EML4*(13)-*ALK*(20)	1659
13	F	40	Histological	Resection	80%	*ALK*	IHC	chr2:42492091-chr2:29446394	*EML4*(6)-*ALK*(20)	349
14	M	57	Histological	Biopsy	70%	*ALK*	IHC	chr2:42491871-chr2:29446394	*EML4*(6)-*ALK*(20)	4401
15	M	55	Histological	Biopsy	60%	*ALK*	IHC	chr2:42543190-chr2:29446463	*EML4*(18)-*ALK*(20)	460
16	F	34	Cytological	Cell-block	60%	*ALK*	IHC	chr2:42552694-chr2:29446394	*EML4*(20)-*ALK*(20)	12,782
17	F	76	Cytological	Cell-block	20%	*ALK*	NGS	chr2:42522656-chr2:29446394	*EML4*(13)-*ALK*(20)	4145
18	F	65	Cytological	Cell-block	40%	*ALK*	FISH	-	Not detected	-
19	M	71	Cytological	Cell-block	20%	*ALK*	FISH	unknown	*ALK* (unknown partner)	-
20	M	51	Histological	Resection	70%	*ALK*	FISH plus IHC	chr2:42492091-chr2:29446394	*EML4*(6)-*ALK*(20)	16,459
21	M	81	Cytological	Cell-block	70%	*ALK*	FISH plus IHC	chr2:42491871-chr2:29446394	*EML4*(6)-*ALK*(20)	6005
22	F	60	Cytological	Cell-block	70%	*ALK*	FISH	chr2:42522656-chr2:29446394	*EML4*(13)-*ALK*(20)	6079
23	M	46	Cytological	Cell-block	40%	*RET*	NGS	chr10:61665880-chr10:43612032	*CCDC6*(1)-*RET*(12)	27,834
24	F	67	Cytological	Cell-block	20%	*ALK*	FISH	-	Not detected	-
25	F	51	Cytological	Cell-block	20%	*ALK*	FISH	unknown	*ALK* (unknown partner)	-
26	F	49	Histological	Resection	70%	*ALK*	FISH plus IHC	chr2:42491871-chr2:29446394	*EML4*(6)-*ALK*(20)	317
27	M	34	Cytological	Cell-block	70%	*ALK*	NGS	chr2:42522656-chr2:29446394	*EML4*(13)-*ALK*(20)	9513
28	F	59	Cytological	Cell-block	40%	*ROS1*	NGS plus FISH plus IHC	chr4:25678324-chr6:117650609	*SLC34A2*(13)-*ROS1*(32)	194,178
29	F	40	Cytological	Cell-block	20%	*ROS1*	FISH	chr1:154142878-chr6:117642559	*TPM3*(8)-*ROS1*(35)	1211
30	M	73	Histological	Resection	80%	*ALK*	FISH plus IHC	chr7:75172170-chr2:29446394	*HIP1*(28)-*ALK*(20)	24,768
31	M	51	Histological	Biopsy	70%	*ALK*	FISH plus IHC	chr2:42543190-chr2:29446394	*EML4*(18)-*ALK*(20)	19,127
32	M	64	Cytological	Cell-block	40%	*ALK*	NGS	-	Not detected	-
33	F	68	Cytological	Cell-block	70%	*ALK*	IHC	chr2:42491871-chr2:29446394	*EML4*(6)-*ALK*(20)	1587
34	M	52	Cytological	Cell-block	20%	*ROS1*	FISH plus IHC	chr5:149784243-chr6:117645578	*CD74*(6)-*ROS1*(34)	5340
35	M	61	Cytological	Cell-block	60%	*RET*	NGS	chr10:32317356-chr10:43612032	*KIF5B*(15)-*RET*(12)	29,390
36	M	61	Cytological	Cell-block	30%	*ALK*	FISH plus IHC	unknown	*ALK* (unknown partner)	-
37	F	25	Histological	Biopsy	30%	*ALK*	NGS	-	Not detected	Failed
38	F	77	Histological	Biopsy	50%	*MET*	NGS	-	*MET* exon 14 skipping	-
39	F	28	Cytological	Cell-block	60%	*ALK*	NGS	chr2:42522656-chr2:29446394	*EML4*(13)-*ALK*(20)	50,661
40	F	46	Histological	Resection	30%	*ALK*	NGS	chr2:42522656-chr2:29446394	*EML4*(13)-*ALK*(20)	2385
41	F	62	Histological	Resection	60%	*MET*	NGS	-	*MET* exon 14 skipping	-
42	M	57	Histological	Resection	60%	*ALK*	NGS	chr2:42522656-chr2:29446394	*EML4*(13)-*ALK*(20)	10,836
43	F	74	Cytological	Cell-block	40%	*RET*	NGS	chr10:61665880-chr10:43612032	*CCDC6*(1)-*RET*(12)	15,342
44	M	59	Histological	Biopsy	20%	*RET*	NGS	chr10:61665880-chr10:43612032	*CCDC6*(1)-*RET*(12)	24,018
45	F	62	Histological	Biopsy	20%	*ALK*	FISH plus IHC	chr2:42552694-chr2:29446394	*EML4*(20)-*ALK*(20)	11,823
46	M	62	Histological	Biopsy	50%	*ALK*	NGS plus IHC	chr2:42472827-chr2:29446394	*EML4*(2)-*ALK*(20)	53,460
47	F	71	Histological	Resection	50%	*MET*	NGS	-	*MET* exon 14 skipping	-
48	M	48	Histological	Biopsy	80%	*ALK*	FISH plus IHC	-	Not detected	Failed

Abbreviations: *ALK*: Anaplastic Lymphoma Kinase; *CCDC6*: Coiled-Coil Domain Containing 6; *CD74*: CD74 Molecule, Major Histocompatibility Complex, Class II Invariant Chain; chr: chromosome; *EML4*: Echinoderm Microtubule Associated Protein Like 4; F: female; FISH: fluorescent in situ hybridization; *HIP1*: Huntingtin Interacting Protein 1; IHC: immunohistochemistry; *KIF5B*: Kinesin Family Member 5B; M: male; *MET*: MET Proto-Oncogene, Receptor Tyrosine Kinase; NGS: next generation sequencing; *RET*: Rearranged During Transfection; *ROS1*: ROS Proto-Oncogene 1, Receptor Tyrosine Kinase; *SLC34A2*: Solute Carrier Family 34 Member 2; *SDC4*: Syndecan 4; *TPM3*: Tropomyosin 3.

## Data Availability

The data presented in this study are available in the manuscript and in the supplementary files.

## Supplementary Materials

The following are available online at https://www.mdpi.com/2072-6694/13/1/139/s1. Supplementary File S1. SiRe fusion panel: design. Supplementary File S2. SiRe fusion panel: software for analysis. Supplementary File S3. SiRe fusion panel: run parameters. Supplementary Table S1. American Type Culture Collection Cell lines used to assess limit of detection and reference range of the SiRe fusion primer pool and relative results obtained with the developed analytical and bioinformatics workflow. Supplementary Table S2. The SiRe fusion results at different dilution points. Supplementary Table S3. Results obtained from FP—MEOH samples. Fused transcripts identified with our NGS approach are highlighted in red. Supplementary Table S4. Results obtained from FP—ETOH samples. The fused transcripts identified with our NGS approach are highlighted in red. Supplementary Table S5. Results obtained from FN—MEOH samples. Supplementary Table S6. Results obtained from FN—ETOH samples. Supplementary Table S7. Fusion positive results obtained from FP—MEOH residual RNA samples after storage for one month at −20 °C. Supplementary Figure S1. Panel A shows the result obtained from RNA extracted from FN-ETOH; Panel B shows the result obtained from cDNA produced from the same sample; Panel C shows the results obtained in RNA extracted from FN-MEOH; Panel D shows the results obtained from cDNA produced from the same sample. Overall, enough quality and the quantity of RNA and relative cDNA was obtained prepare libraries for next-generation sequencing analysis (NGS) by using our SiRe^®^ fusion panel (Department of Public Health, University of Naples Federico II). Supplementary Figure S2. Panel A shows the results obtained from RNA extracted from FP-ETOH; Panel B shows the results obtained from cDNA produced from the same sample; Panel C shows the result obtained from RNA extracted from FP-MEOH; Panel D shows the results obtained from cDNA produced from the same sample. Overall, the quality and the quantity of RNA and relative cDNA were adequate to prepare libraries for next-generation sequencing analysis (NGS) by using our SiRe^®^ fusion panel (Department of Public Health, University of Naples Federico II).
