# Supplementary material for: RNA-Based Assay for Next-Generation Sequencing of Clinically Relevant Gene Fusions in Non-Small Cell Lung Cancer
Source: Cancers (Basel). 2021 Jan 4;13(1):139. doi: 10.3390/cancers13010139 (PMC7796105; doi:10.3390/cancers13010139)
Supplement: Supplementary file 1 [file cancers-13-00139-s001.zip › Supplementary files/Supplementary File 3.pdf]

Run Summary

430 M Total Bases

114 Key Signal

91% ISP Loading

ISP Density

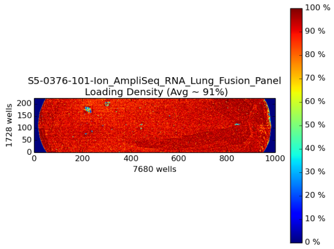

4,160,598 Total Reads

37% Usable Reads

ISP Summary

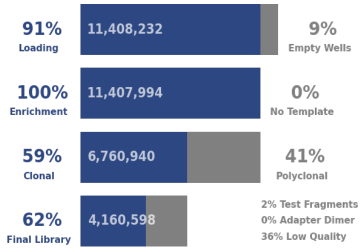

103 bp Mean

94 bp Median

95 bp Mode

Read Length

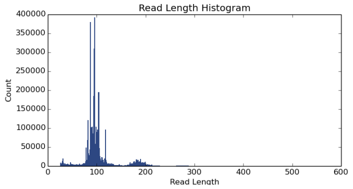

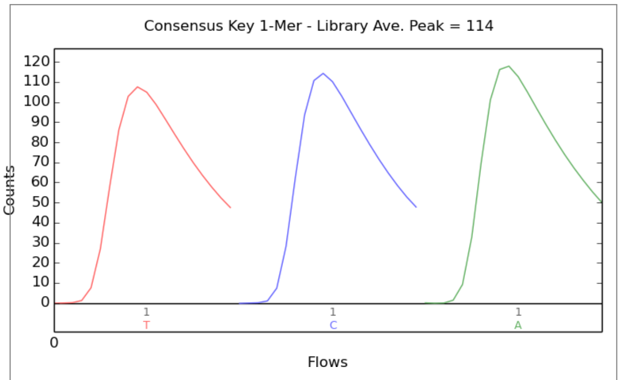

**Addressable Wells**	**12,530,194**	
With ISPs	11,408,232	91.0%
Live	11,407,994	100.0%
Test Fragment	114,012	01.0%
Library	11,293,982	99.0%

**Library ISPs**	**11,293,982**	
Filtered: Polyclonal	4,647,054	41.1%
Filtered: Low Quality	2,477,673	21.9%
Filtered: Adapter Dimer	8,657	00.1%
**Final Library ISPs**	**4,160,598**	**36.8%**

Barcode Name	Sample	Bases	$\geq Q20$	Reads	Mean Read Length	Read Length Histogram
No barcode	none	23,286,386	18,786,252	217,568	107 bp	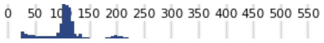
IonCode_0117	Fusion18B3385	32,494,711	29,106,231	303,128	107 bp	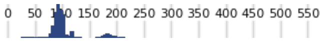
IonCode_0118	Fusion18I895	17,704,604	16,210,780	185,939	95 bp	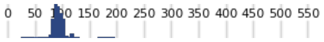
IonCode_0119	Fusion18B2494	14,676,687	13,507,031	159,090	92 bp	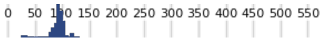
IonCode_0120	Fusion18I2993	15,485,930	14,069,004	156,947	98 bp	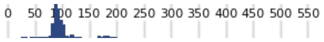
IonCode_0121	Fusion19B122	32,790,809	29,655,099	302,515	108 bp	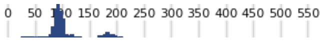
IonCode_0122	Fusion_19_B_2	21,345,329	19,593,380	214,921	99 bp	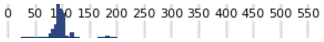
IonCode_0123	Fusion_20_F508	25,827,780	23,585,994	217,258	118 bp	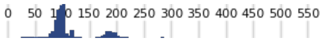

iontorrent by Thermo Fisher Scientific

IonCode_0124	Fusion_ 19_57	29,610,448	27,100,888	262,757	112 bp	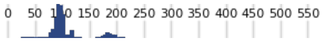
IonCode_0125	Fusion_18_352	58,490,079	52,884,802	500,331	116 bp	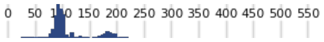
IonCode_0126	Fusion_17_8717	43,291,691	39,337,018	406,457	106 bp	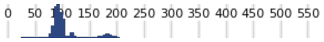
IonCode_0127	Fusion_15_3258	25,927,547	23,880,014	274,466	94 bp	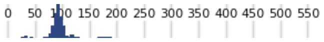
IonCode_0128	Fusion_19_571	37,756,521	34,615,439	398,107	94 bp	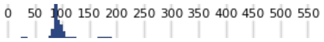
IonCode_0129	Fusion_18_127	19,999,745	18,234,791	201,903	99 bp	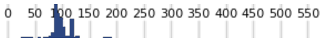
IonCode_0130	Fusion_4210	11,318,222	10,272,246	131,172	86 bp	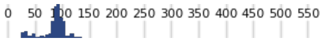
IonCode_0131	Fusion_17_7995	9,314,877	8,579,374	111,430	83 bp	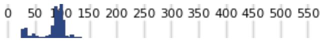
IonCode_0132	Fusion_14736	10,891,724	9,941,941	116,583	93 bp	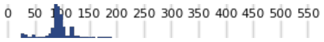

Test Fragment	Reads	Percent 50AQ17	Read Length Histogram
**TF_1**	**29,667**	**94**	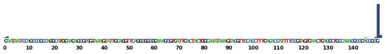

iontorrent by Thermo Fisher Scientific

Alignment Summary *(aligned to SiRe_fusion)*

345 M	27255.8X
Total Alignment Bases	Average Coverage Depth of Reference

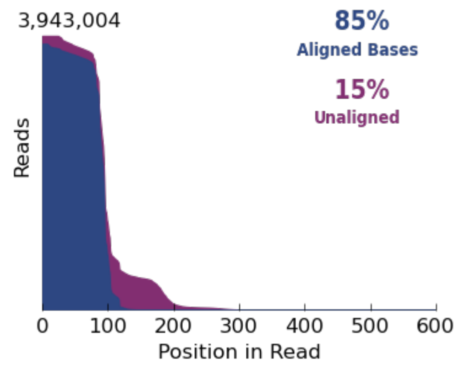

	Count	%
**Total Reads**	3,943,004	–
**Aligned Reads**	3,829,000	97.1%
**Unaligned Reads**	114,004	2.9%

98.8%

Mean Raw Accuracy 1x

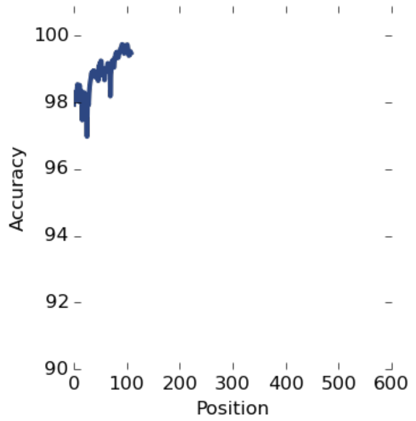

Alignment Quality

	AQ17	AQ20	Perfect
**Total Number of Bases [Mbp]**	301 M	260 M	251 M
**Mean Length [bp]**	87	80	78
**Longest Alignment [bp]**	154	154	153
**Mean Coverage Depth**	23834.5	20551.2	19842.9

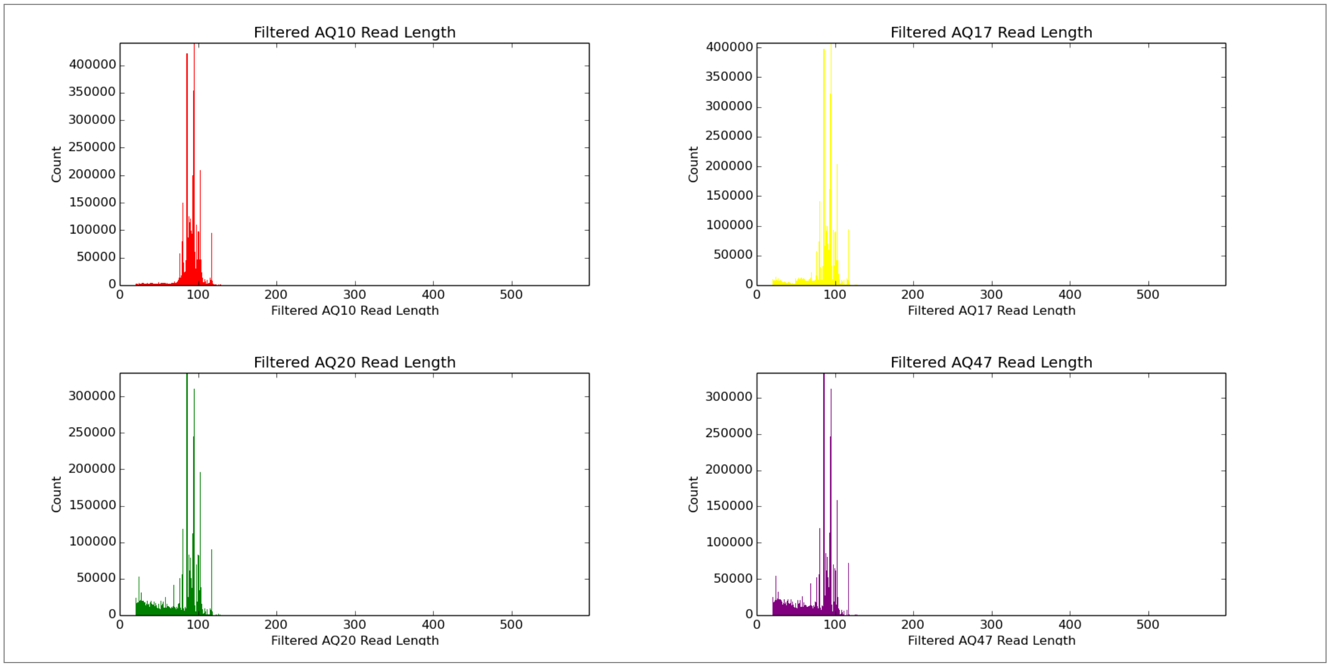
Filtered AQ10 Read Length
Count
Filtered AQ10 Read Length
Filtered AQ17 Read Length
Count
Filtered AQ17 Read Length
Filtered AQ20 Read Length
Count
Filtered AQ20 Read Length
Filtered AQ47 Read Length
Count
Filtered AQ47 Read Length

coverageAnalysis

Library type: AmpliSeq RNA
Target regions: WG00250_ExtendedLungFusion_Designed

Barcode Name	Sample	Mapped Reads	On Target
IonCode_0117	Fusion_18_B_3385	299,664	99.62%
IonCode_0118	Fusion_18_I_895	180,976	99.25%
IonCode_0119	Fusion_18_B_2494	154,359	97.95%
IonCode_0120	Fusion_18_I_2993	153,463	99.12%
IonCode_0121	Fusion_19_B_122	298,962	99.68%
IonCode_0122	Fusion_19_B_2	210,894	99.09%
IonCode_0123	Fusion_20_F_508	215,587	99.82%
IonCode_0124	Fusion_Pietoso	260,914	99.77%
IonCode_0125	Fusion_Caporaso	491,093	99.58%
IonCode_0126	Fusion_Ponticelli	391,648	99.12%
IonCode_0127	Fusion_Frallicciardi	270,813	98.69%
IonCode_0128	Fusion_Abbate	393,114	99.20%
IonCode_0129	Fusion_Canto	199,035	99.07%
IonCode_0130	Fusion_4210	111,104	93.02%
IonCode_0131	Fusion_17_7995	96,590	91.45%
IonCode_0132	Fusion_14736	100,784	95.17%

1 | 5 items per page | 1 - 16 of 16 items

iontorrent by Thermo Fisher Scientific

Analysis Details

**Run Name**	R_2020_06_16_09_02_48_user_S5-0376-101-Ion_AmpliSeq_RNA_Lung_Fusion_Panel
**Run Date**	June 16, 2020, 9:04 a.m.
**Run Flows**	850
**Projects**	
**Sample**	Fusion_Abbate, Fusion_14736, Fusion_18_B_3385, Fusion_Canto, Fusion_Ponticelli, Fusion_4210, Fusion_19_B_122, Fusion_Pietoso, Fusion_18_I_2993, Fusion_Frallicciardi, Fusion_Caporaso, Fusion_18_I_895, Fusion_18_B_2494, Fusion_19_B_2, Fusion_20_F_508, Fusion_17_7995
**Reference**	
**Instrument**	S5-0376
**Operation Mode**	Customer mode
**Flow Order**	TACGTACGTCTGAGCATCGATCGATGTACAGC
**Library Key**	TCAG
**TF Key**	ATCG
**Chip Barcode**	DAGA02297
**Chip Check**	Passed
**Chip Type**	520
**Chip Data**	tiled
**Chip Lot Number**	Q0RC69
**Chip Wafer**	23
**Barcode Set**	IonCode Barcodes 1-32
**Analysis Name**	Auto_user_S5-0376-101-Ion_AmpliSeq_RNA_Lung_Fusion_Panel_341
**Analysis Date**	June 16, 2020, 4:02 p.m.
**Analysis Flows**	0
**runID**	CF5Q0
**BeadFind Args**	justBeadFind –args-json /opt/ion/config/args_520_beadfind.json
**Analysis Args**	Analysis –args-json /opt/ion/config/args_520_analysis.json
**Pre-BaseCaller**	BaseCaller –trim-qual-cutoff 15 –barcode-filter-minreads 10 –phasing-residual-filter=2.0 –wells-normalization on
**Calibration Args**	Calibration –num-calibration-regions 1,1
**BaseCaller Args**	BaseCaller –trim-qual-cutoff 15 –barcode-filter-minreads 10 –phasing-residual-filter=2.0 –num-unfiltered 1000 –barcode-filter-postpone 1 –qual-filter true –qual-filter-slope 0.040 –qual-filter-offset 1.0 –wells-normalization on
**Alignment Args**	tmap mapall ... stage1 map4
**IonStats Args**	ionstats alignment
**Analysis Parameters**	default

iontorrent by Thermo Fisher Scientific

Chef Summary

Chef Template Prep Information:

**Chef Last Updated**	June 16, 2020, 8:43 a.m.
**Chef Instrument Name**	CHEF00839
**Chef Operation Mode**	Customer Mode
**Sample Position**	1
**Tip Rack Barcode**	48B1400D8
**Chip Type 1**	520v1
**Chip Type 2**	S500
**Chip Expiration 1**	None
**Chip Expiration 2**	None
**Templating Kit Type**	Ion 510
amp; Ion 520	amp; Ion 530 Kit-Chef
**Chef Flexible Workflow**	
**Reagent Expiration**	200331
**Reagent Lot Number**	2070211
**Reagent Part Number**	A34018C
**Reagent Cartridge Serial Number**	None
**Solution Lot Number**	2065570
**Solution Part Number**	A27754C
**Templating Protocol Planned**	(use instrument default)
**Solution Cartridge Serial Number**	None
**Solution Expiration**	200331
**Templating Protocol Executed**	(use instrument default)
**Chef Script Version**	905
**Chef Package Version**	IC.5.12.0
**Start Time**	June 15, 2020, 12:19 p.m.
**Completion Time**	June 16, 2020, 8:43 a.m.

S5 Consumables Summary

**Chip Type**	520v1
**Chip Barcode**	DAGA02297

Product Description	Part Number	Lot Number	Exp. Date	Remaining Uses
Ion S5 Cleaning Solution	100031096	2049959	2019/12/31	5
Ion S5 Sequencing Reagent	INS1012841B	2049947	2020/01/31	1
Ion S5 Wash Solution	100031091B	2058327	2020/02/29	1

iontorrent by Thermo Fisher Scientific

Software Version

**Torrent_Suite**	5.12.0
**host**	tsvm
**ion-analysis**	5.12.11-1
**ion-dbreports**	5.12.35-1
**ion-gpu**	5.12.0-1
**ion-pipeline**	5.12.11-1
**ion-torrentpy**	5.12.8-1
**ion-torrentr**	5.12.10-1
**S5 Script**	0.1.31
**LiveView**	2514
**DataCollect**	3738
**OIA**	51206
**OS**	35
**Graphics**	134
**Ion_Chef**	IC.5.12.0

Run Summary

524 M Total Bases | **128** Key Signal

93% ISP Loading

ISP Density

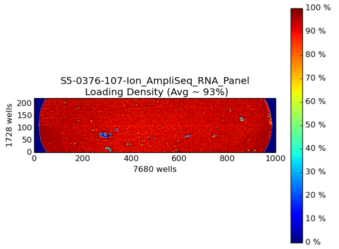

5,660,804 Total Reads

49% Usable Reads

ISP Summary

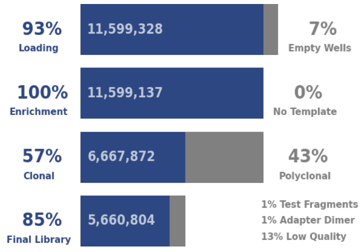

93 bp Mean | **93 bp** Median | **95 bp** Mode

Read Length

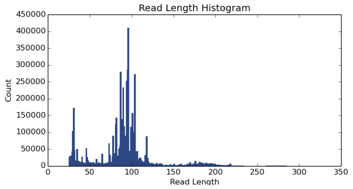

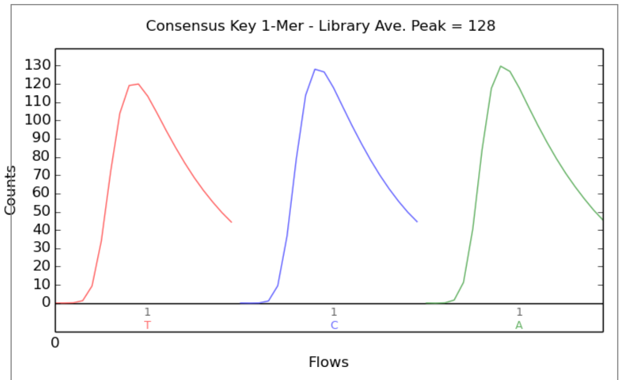

**Addressable Wells**	**12,530,194**	
With ISPs	11,599,328	92.6%
Live	11,599,137	100.0%
Test Fragment	92,235	00.8%
Library	11,506,902	99.2%

**Library ISPs**	**11,506,902**	
Filtered: Polyclonal	4,931,265	42.9%
Filtered: Low Quality	874,809	07.6%
Filtered: Adapter Dimer	40,024	00.3%
**Final Library ISPs**	**5,660,804**	**49.2%**

Barcode Name	Sample	Bases	$\geq Q20$	Reads	Mean Read Length	Read Length Histogram
No barcode	none	25,135,011	22,608,959	239,194	105 bp	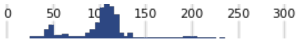
IonCode_0117	FUSION16C_5752	32,167,326	30,068,815	361,649	88 bp	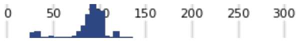
IonCode_0118	FUSION13C_5007	25,337,140	23,870,898	329,494	76 bp	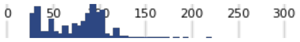
IonCode_0119	FUSION11C_8597	9,064,261	8,526,987	187,641	48 bp	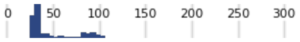
IonCode_0120	FUSION11S_5960	50,568,773	47,526,040	485,126	104 bp	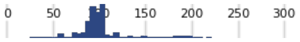
IonCode_0121	FUSION18C_482	34,894,704	32,707,467	372,192	93 bp	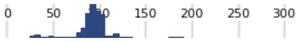
IonCode_0122	FUSION11C_9307	31,313,401	29,344,945	326,568	95 bp	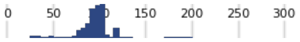
IonCode_0123	FUSION20C_374	50,057,995	46,099,437	390,759	128 bp	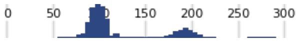

iontorrent by Thermo Fisher Scientific

IonCode_0124	FUSION15C4638	12,865,682	12,202,244	170,404	75 bp	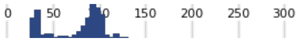
IonCode_0125	FUSION13C2416	16,765,306	15,684,592	256,212	65 bp	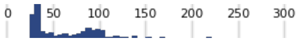
IonCode_0126	FUSION14I2731	26,720,082	25,030,441	341,244	78 bp	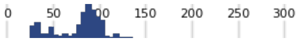
IonCode_0127	FUSION10C2977	48,192,941	44,937,245	461,455	104 bp	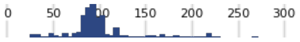
IonCode_0128	FUSION20C1459	66,593,968	61,805,458	644,418	103 bp	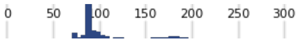
IonCode_0129	FUSION15C416B	28,511,208	26,412,449	308,298	92 bp	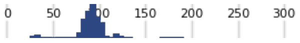
IonCode_0130	FUSION17I5356	26,496,025	24,848,973	306,868	86 bp	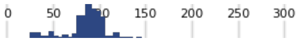
IonCode_0131	FUSION18I2137	32,723,835	30,706,310	351,711	93 bp	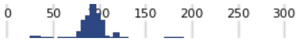
IonCode_0132	FUSION14C4583C	7,049,194	6,705,216	127,519	55 bp	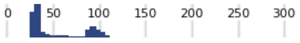

Test Fragment	Reads	Percent 50AQ17	Read Length Histogram
**TF_1**	**29,191**	**96**	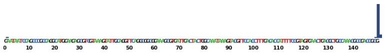

Alignment Summary *(aligned to hg19 AmpliSeq Transcriptome v1.1)*

391 M	5.8X
Total Alignment Bases	Average Coverage Depth of Reference

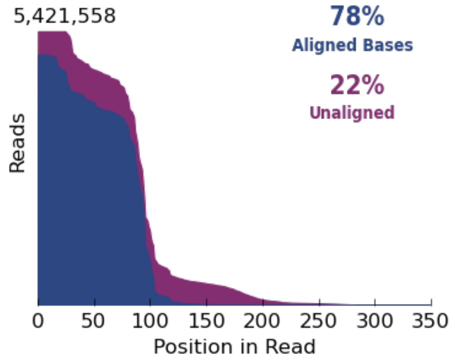

	Count	%
**Total Reads**	5,421,558	–
**Aligned Reads**	4,960,816	91.5%
**Unaligned Reads**	460,742	8.5%

98.3%

Mean Raw Accuracy 1x

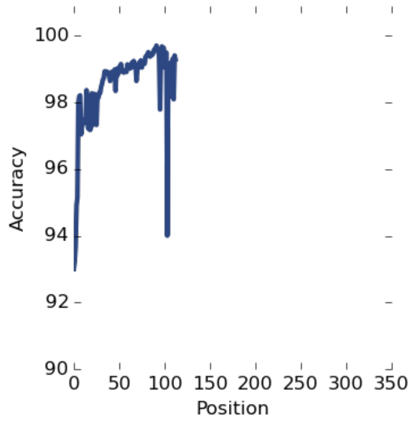

Alignment Quality

	AQ17	AQ20	Perfect
**Total Number of Bases [Mbp]**	327 M	283 M	272 M
**Mean Length [bp]**	84	77	75
**Longest Alignment [bp]**	297	297	283
**Mean Coverage Depth**	4.9	4.2	4.0

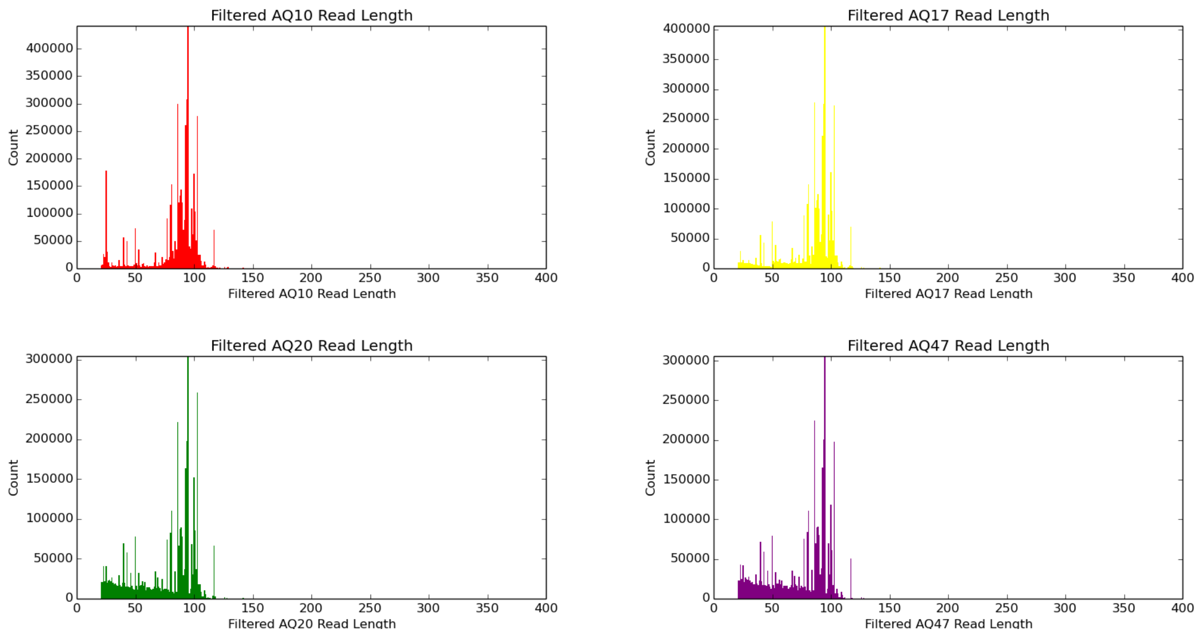

iontorrent by Thermo Fisher Scientific

coverageAnalysis

All barcodes had invalid target regions specified.

Analysis Details

**Run Name**	R_2020_07_02_13_08_04_user_S5-0376-107-Ion_AmpliSeq_RNA_Panel
**Run Date**	July 2, 2020, 1:09 p.m.
**Run Flows**	500
**Projects**	
**Sample**	FUSION_13_C_5007A, FUSION_15_C_4638, FUSION_20_C_374B, FUSION_10_C_2977, FUSION_14_I_2731A3, FUSION_20_C_1459D, FUSION_16_C_5752A, FUSION_18_I_2137A1, FUSION_13_C_2416C, FUSION_14_C_4583C, FUSION_15_C_416B, FUSION_11_C_8597, FUSION_11_S_5960, FUSION_11_C_9307B, FUSION_18_C_482B, FUSION_17_I_5356A2
**Reference**	
**Instrument**	S5-0376
**Operation Mode**	Customer mode
**Flow Order**	TACGTACGTCTGAGCATCGATCGATGTACAGC
**Library Key**	TCAG
**TF Key**	ATCG
**Chip Barcode**	DAGB02050
**Chip Check**	Passed
**Chip Type**	520
**Chip Data**	tiled
**Chip Lot Number**	Q0SS79
**Chip Wafer**	18
**Barcode Set**	IonCode Barcodes 1-32
**Analysis Name**	reanalisi
**Analysis Date**	Dec. 22, 2020, 7:20 p.m.
**Analysis Flows**	0
**runID**	NDRE0
**BeadFind Args**	justBeadFind –args-json /opt/ion/config/args_520_beadfind.json
**Analysis Args**	Analysis –args-json /opt/ion/config/args_520_analysis.json
**Pre-BaseCaller**	BaseCaller –trim-qual-cutoff 15 –barcode-filter-minreads 10 –phasing-residual-filter=2.0 –wells-normalization on
**Calibration Args**	Calibration –num-calibration-regions 1,1
**BaseCaller Args**	BaseCaller –trim-qual-cutoff 15 –barcode-filter-minreads 10 –phasing-residual-filter=2.0 –num-unfiltered 1000 –barcode-filter-postpone 1 –qual-filter true –qual-filter-slope 0.040 –qual-filter-offset 1.0 –wells-normalization on
**Alignment Args**	tmap mapall ... stage1 map4
**IonStats Args**	ionstats alignment
**Analysis Parameters**	default

Chef Summary

Chef Template Prep Information:

**Chef Last Updated**	July 2, 2020, 8:35 a.m.
**Chef Instrument Name**	CHEF00839
**Chef Operation Mode**	Customer Mode
**Sample Position**	2
**Tip Rack Barcode**	48B1400DF
**Chip Type 1**	520v1
**Chip Type 2**	520v1
**Chip Expiration 1**	None
**Chip Expiration 2**	None
**Templating Kit Type**	Ion 510
amp; Ion 520	amp; Ion 530 Kit-Chef
**Chef Flexible Workflow**	
**Reagent Expiration**	200331
**Reagent Lot Number**	2070211
**Reagent Part Number**	A34018C
**Reagent Cartridge Serial Number**	None
**Solution Lot Number**	2065570
**Solution Part Number**	A27754C
**Templating Protocol Planned**	Chef Protocol - 200 bp
**Solution Cartridge Serial Number**	None
**Solution Expiration**	200331
**Templating Protocol Executed**	Chef Protocol - 200 bp
**Chef Script Version**	905
**Chef Package Version**	IC.5.12.0
**Start Time**	July 1, 2020, 3:04 p.m.
**Completion Time**	July 2, 2020, 8:35 a.m.

S5 Consumables Summary

**Chip Type**	520v1
**Chip Barcode**	DAGB02050

Product Description	Part Number	Lot Number	Exp. Date	Remaining Uses
Ion S5 Cleaning Solution	100031096	2049959	2019/12/31	2
Ion S5 Sequencing Reagent	INS1012841B	2049947	2020/01/31	1
Ion S5 Wash Solution	100031091B	2044840	2019/12/31	1

iontorrent by Thermo Fisher Scientific

Software Version

**Torrent_Suite**	5.12.0
**host**	tsvm
**ion-analysis**	5.12.11-1
**ion-dbreports**	5.12.35-1
**ion-gpu**	5.12.0-1
**ion-pipeline**	5.12.11-1
**ion-torrentpy**	5.12.8-1
**ion-torrentr**	5.12.10-1
**S5 Script**	0.1.31
**LiveView**	2514
**DataCollect**	3738
**OIA**	51206
**OS**	35
**Graphics**	134
**Ion_Chef**	IC.5.12.0

iontorrent by Thermo Fisher Scientific

Run Summary

626 M Total Bases | **130** Key Signal

94% ISP Loading

ISP Density

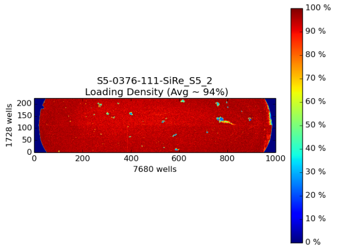

6,407,651 Total Reads

55% Usable Reads

ISP Summary

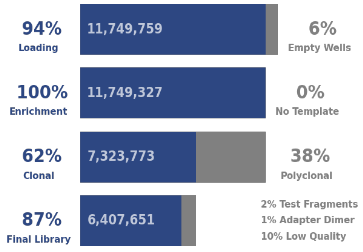

98 bp Mean | **94 bp** Median | **95 bp** Mode

Read Length

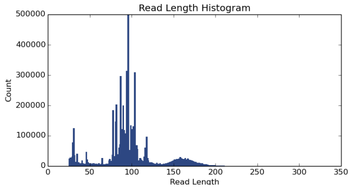

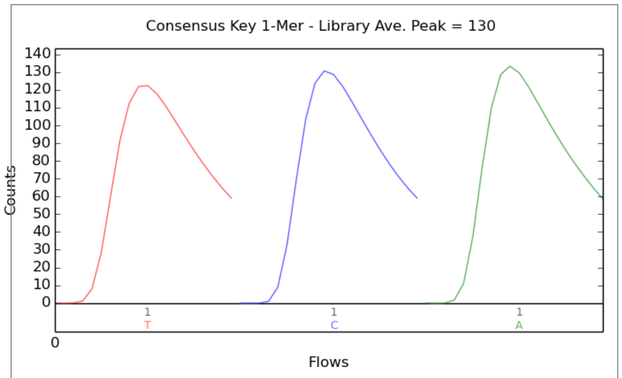

**Addressable Wells**	**12,530,194**	
With ISPs	11,749,759	93.8%
Live	11,749,327	100.0%
Test Fragment	130,985	01.1%
Library	11,618,342	98.9%

**Library ISPs**	**11,618,342**	
Filtered: Polyclonal	4,425,554	38.1%
Filtered: Low Quality	740,623	06.4%
Filtered: Adapter Dimer	44,514	00.4%
**Final Library ISPs**	**6,407,651**	**55.2%**

Barcode Name	Sample	Bases	$\geq Q20$	Reads	Mean Read Length	Read Length Histogram
No barcode	none	28,557,791	25,817,138	252,793	112 bp	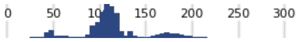
IonCode_0117	FUSION16C1355	46,701,436	42,959,196	516,927	90 bp	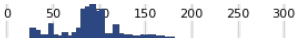
IonCode_0118	FUSION17C3735	25,371,461	23,622,657	307,416	82 bp	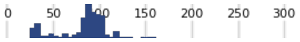
IonCode_0119	FUSION19C5303	122,118,381	111,309,073	1,099,371	111 bp	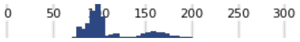
IonCode_0120	FUSION15C3437	14,111,335	13,142,458	227,636	61 bp	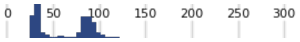
IonCode_0121	FUSION11I6906	12,274,906	11,484,596	192,366	63 bp	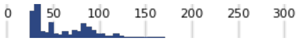
IonCode_0122	FUSION20I2504	63,355,433	56,456,056	538,211	117 bp	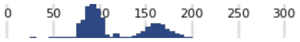
IonCode_0123	FUSION15C5484	38,299,723	36,259,745	413,877	92 bp	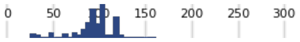

IonCode_0124	FUSION10_I133_14	20,047,001	18,895,169	249,408	80 bp	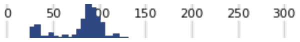
IonCode_0125	FUSION19_I10_8_01	48,487,591	44,064,260	436,924	110 bp	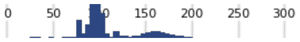
IonCode_0126	FUSION20_I219_	_39,822,230	36,994,926	418,621	95 bp	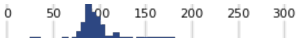
IonCode_0127	FUSION20_C8_03_B	48,802,023	44,918,992	502,476	97 bp	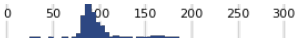
IonCode_0128	FUSION18_I91_3_0	19,018,879	18,030,470	221,075	86 bp	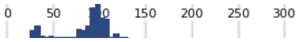
IonCode_0129	FUSION18_I30_4_	14,313,847	13,236,148	181,476	78 bp	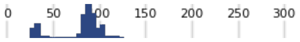
IonCode_0130	FUSION19_I58_7_9B3	35,860,331	33,544,606	364,420	98 bp	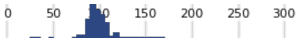
IonCode_0131	FUSION19_I11_1_40	44,831,921	40,532,297	403,814	111 bp	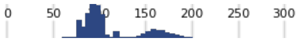
IonCode_0132	FUSION15_I49_9_5	3,782,520	3,558,085	80,789	46 bp	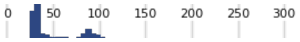

Test Fragment	Reads	Percent 50AQ17	Read Length Histogram
**TF_1**	**60,187**	**94**	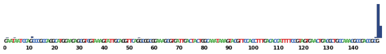

iontorrent by Thermo Fisher Scientific

Alignment Summary *(aligned to hg19 AmpliSeq Transcriptome v1.1)*

474 M
Total Alignment Bases

7.1X
Average Coverage Depth of Reference

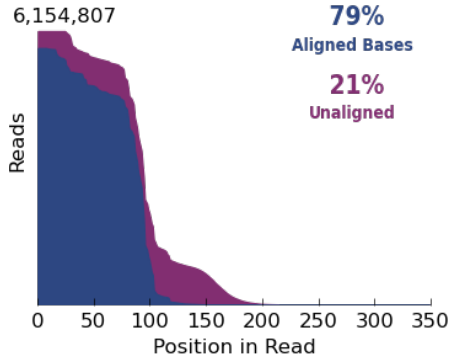

	Count	%
**Total Reads**	6,154,807	–
**Aligned Reads**	5,772,340	93.8%
**Unaligned Reads**	382,467	6.2%

98.7%
Mean Raw Accuracy 1x

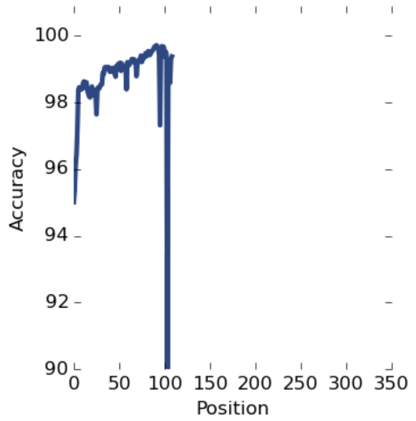

Alignment Quality

	AQ17	AQ20	Perfect
**Total Number of Bases [Mbp]**	413 M	364 M	353 M
**Mean Length [bp]**	84	78	76
**Longest Alignment [bp]**	221	209	203
**Mean Coverage Depth**	6.2	5.4	5.2

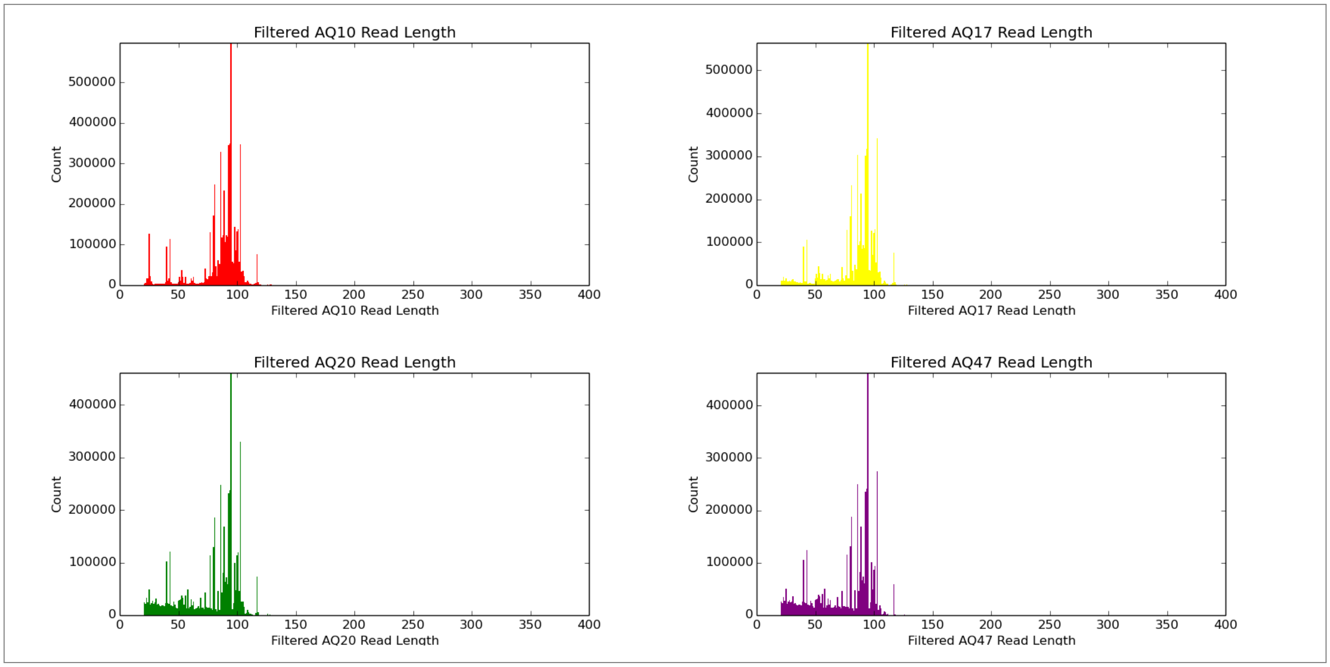

iontorrent by Thermo Fisher Scientific

coverageAnalysis

All barcodes had invalid target regions specified.

Analysis Details

**Run Name**	R_2020_07_14_12_38_52_user_S5-0376-111-SiRe_S5_2
**Run Date**	July 14, 2020, 12:40 p.m.
**Run Flows**	500
**Projects**	
**Sample**	FUSION_16_C_1355B, FUSION_15_I_4995, FUSION_19_C_5303_B, FUSION_18_I_9130_A1, FUSION_20_I_219_A2, FUSION_18_I_304B1, FUSION_19_I_11140B3, FUSION_11_I_6906_B, FUSION_15_C_3437_A, FUSION_19_I_10801A1, FUSION_19_I_5879B3, FUSION_10_I_13314C3, FUSION_15_C_5484B, FUSION_20_C_803B, FUSION_17_C_3735, FUSION_20_I_2504A1
**Reference**	
**Instrument**	S5-0376
**Operation Mode**	Customer mode
**Flow Order**	TACGTACGTCTGAGCATCGATCGATGTACAGC
**Library Key**	TCAG
**TF Key**	ATCG
**Chip Barcode**	DAGB02071
**Chip Check**	Passed
**Chip Type**	520
**Chip Data**	tiled
**Chip Lot Number**	Q0SS79
**Chip Wafer**	18
**Barcode Set**	IonCode Barcodes 1-32
**Analysis Name**	reanalisi_trascri
**Analysis Date**	July 15, 2020, 10:02 a.m.
**Analysis Flows**	0
**runID**	B4869
**BeadFind Args**	justBeadFind –args-json /opt/ion/config/args_520_beadfind.json
**Analysis Args**	Analysis –args-json /opt/ion/config/args_520_analysis.json
**Pre-BaseCaller**	BaseCaller –trim-qual-cutoff 15 –barcode-filter-minreads 10 –phasing-residual-filter=2.0 –wells-normalization on
**Calibration Args**	Calibration –num-calibration-regions 1,1
**BaseCaller Args**	BaseCaller –trim-qual-cutoff 15 –barcode-filter-minreads 10 –phasing-residual-filter=2.0 –num-unfiltered 1000 –barcode-filter-postpone 1 –qual-filter true –qual-filter-slope 0.040 –qual-filter-offset 1.0 –wells-normalization on
**Alignment Args**	tmap mapall ... stage1 map4
**IonStats Args**	ionstats alignment
**Analysis Parameters**	default

iontorrent by Thermo Fisher Scientific

Chef Summary

Chef Template Prep Information:

**Chef Last Updated**	July 14, 2020, 8:33 a.m.
**Chef Instrument Name**	CHEF00839
**Chef Operation Mode**	Customer Mode
**Sample Position**	2
**Tip Rack Barcode**	48B140103
**Chip Type 1**	520v1
**Chip Type 2**	520v1
**Chip Expiration 1**	None
**Chip Expiration 2**	None
**Templating Kit Type**	Ion 510
amp; Ion 520	amp; Ion 530 Kit-Chef
**Chef Flexible Workflow**	
**Reagent Expiration**	200331
**Reagent Lot Number**	2070211
**Reagent Part Number**	A34018C
**Reagent Cartridge Serial Number**	None
**Solution Lot Number**	2065570
**Solution Part Number**	A27754C
**Templating Protocol Planned**	Chef Protocol - 200 bp
**Solution Cartridge Serial Number**	None
**Solution Expiration**	200331
**Templating Protocol Executed**	Chef Protocol - 200 bp
**Chef Script Version**	905
**Chef Package Version**	IC.5.12.0
**Start Time**	July 13, 2020, 1:28 p.m.
**Completion Time**	July 14, 2020, 8:33 a.m.

S5 Consumables Summary

**Chip Type**	520v1
**Chip Barcode**	DAGB02071

Product Description	Part Number	Lot Number	Exp. Date	Remaining Uses
Ion S5 Cleaning Solution	100031096	2049959	2019/12/31	4
Ion S5 Sequencing Reagent	INS1012841B	2049947	2020/01/31	1
Ion S5 Wash Solution	100031091B	2044840	2019/12/31	1

Software Version

**Torrent_Suite**	5.12.0
**host**	tsvm
**ion-analysis**	5.12.11-1
**ion-dbreports**	5.12.35-1
**ion-gpu**	5.12.0-1
**ion-pipeline**	5.12.11-1
**ion-torrentpy**	5.12.8-1
**ion-torrentr**	5.12.10-1
**S5 Script**	0.1.31
**LiveView**	2514
**DataCollect**	3738
**OIA**	51206
**OS**	35
**Graphics**	134
**Ion_Chef**	IC.5.12.0
