# Supplementary material for: RNA-Based Assay for Next-Generation Sequencing of Clinically Relevant Gene Fusions in Non-Small Cell Lung Cancer
Source: Cancers (Basel). 2021 Jan 4;13(1):139. doi: 10.3390/cancers13010139 (PMC7796105; doi:10.3390/cancers13010139)
Supplement: Supplementary file 1 [file cancers-13-00139-s001.zip › Supplementary files/Supplementary Table 1.docx]

**Supplementary Table 1**. American Type Culture Collection Cell lines used to assess limit of detection and reference range of SiRe fusion primers pool and relative results obtained by using the developed analytical and bioinformatics workflow.

| **Cell line** | **Locus** | **Type** | **Filter** | **Genes (exons)** | **Reads count** | **Detection** |
| --- | --- | --- | --- | --- | --- | --- |
| H3122 | chr2:42522656 –  chr2:29446394 | Fusion | PASS | *EML4*(13) – *ALK*(20) | 13235 | Present |
| H2228 | chr2:42491871 -  chr2:29446394 | Fusion | PASS | *EML4*(6) - *ALK*(20) | 2007 | Present |
| HS746T |  |  | PASS | *MET* 14 exon skipping |  | Present |
| H596 |  |  | PASS | *MET* 14 exon skipping |  | Present |
| HCC78 | chr4:25665952 -  chr6:117650609 | Fusion | PASS | *SLC34A2*(4) - *ROS1*(32) | 12271 | Present |
| LC2ad | chr10:61665880 -  chr10:43612032 | Fusion | PASS | *CCDC6*(1) - *RET*(12) | 26725 | Present |
| EBC-1 |  |  | PASS |  |  | None |
| SUDHL-1 | chr2:29551347  - chr2:29430138 | Fusion | PASS | Unknown-*ALK* | 0.430666 | Present |
| NTRK1cl | chr1:154142878 -  chr1:156844363 | Fusion | PASS | *TPM3*(8) - *NTRK1*(10) | 227248 | Present-Non-Targeted |

Abbreviations: *ALK*: Anaplastic Lymphoma Kinase; *CCDC6*: Coiled-Coil Domain Containing 6; chr: chromosome; *EML4*: Echinoderm Microtubule-Associated Protein-Like 4; *MET*: MET Proto-Oncogene, Receptor Tyrosine Kinase; *NTRK1*: Neurotrophic Receptor Tyrosine Kinase 1; *RET*: Rearranged During Transfection; *ROS1*: ROS Proto-Oncogene 1, Receptor Tyrosine Kinase; *SLC34A2*: Solute Carrier Family 34 Member 2; *TPM3*: Tropomyosin 3.
