# Supplementary material for: RNA-Based Assay for Next-Generation Sequencing of Clinically Relevant Gene Fusions in Non-Small Cell Lung Cancer
Source: Cancers (Basel). 2021 Jan 4;13(1):139. doi: 10.3390/cancers13010139 (PMC7796105; doi:10.3390/cancers13010139)
Supplement: Supplementary file 1 [file cancers-13-00139-s001.zip › Supplementary files/Supplementary Table 2.docx]

**Supplementary Table 2**. The SiRe fusion results at different dilution points.

| ng/µl **(cell line mix)** | **Filter** | **Fusion Detected** | **Splicing Detected** |
| --- | --- | --- | --- |
| 20.0 | PASS | 6/6 | 1/1 |
| 10.0 | PASS | 6/6 | 1/1 |
| 2.0 | PASS | 6/6 | 1/1 |
| 0.5 | PASS | n.a. | n.a. |
| 0.1 | PASS | n.a. | n.a |

Abbreviation: n.a.: not assessed.
