# Supplementary material for: RNA-Based Assay for Next-Generation Sequencing of Clinically Relevant Gene Fusions in Non-Small Cell Lung Cancer
Source: Cancers (Basel). 2021 Jan 4;13(1):139. doi: 10.3390/cancers13010139 (PMC7796105; doi:10.3390/cancers13010139)
Supplement: Supplementary file 1 [file cancers-13-00139-s001.zip › Supplementary files/Supplementary Table 3.docx]

**Supplementary Table 3.** Results obtained on FP – MEOH sample. the fused transcripts identified by using our NGS approach are highlighted in red.

| Locus | Type | Filter | Genes  (exons) | Read counts | Detection |
| --- | --- | --- | --- | --- | --- |
| chr6:170871321 | EXPR_CON^-^ | PASS | *TBP* | 52448 | Present |
| chr12:53585787 | EXPR_CON^-^ | PASS | *ITGB7* | 10874 | Present |
| chr8:128751265 | EXPR_CON^-^ | PASS | *MYC* | 163240 | Present |
| chr11:118960975 | EXPR_CON^-^ | PASS | *HMBS* | 87238 | Present |
| chr1:156104319 | EXPR_CON^-^ | PASS | *LMNA* | 174150 | Present |
| chr4:25665952 –  chr6:117650609 | FUSION | PASS | *SLC34A2*(4) - *ROS1*(32) | 33224 | Present |
| chr10:61665880 –  chr10:43612032 | FUSION | PASS | *CCDC6*(1) - *RET*(12) | 31148 | Present |
| chr2:42522656 –  chr2:29446394 | FUSION | PASS | *EML4*(10) - *ALK*(20) | 61 | Present |
| chr4:25665952 –  chr6:117645578 | FUSION | PASS | *SLC34A2*(4) - *ROS1*(34) | 402 | Present |
| chr1:154142878 –  chr1:156844363 | FUSION | PASS | *TPM3*(8) - *NTRK1*(10) | 1161 | Present-  Non-Targeted |

Abbreviations: *ALK*: Anaplastic Lymphoma Kinase; *CCDC6*: Coiled-Coil Domain Containing 6; chr: chromosome; *EML4*: Echinoderm Microtubule-Associated Protein-Like 4; FP: fusion positive; *HMBS*: Hydroxymethylbilane Synthase; *ITGB7*: Integrin Subunit Beta 7; *LMNA*: Lamin A/C; MEOH: methanol; *NTRK1*: Neurotrophic Receptor Tyrosine Kinase 1; *TBP*: TATA-Box Binding Protein; *MYC*: MYC Proto-Oncogene, BHLH Transcription Factor; *RET*: Rearranged During Transfection;  *ROS1*: ROS Proto-Oncogene 1, Receptor Tyrosine Kinase; *SLC34A2*: Solute Carrier Family 34 Member 2; *TPM3*: Tropomyosin 3.
