# Supplementary material for: RNA-Based Assay for Next-Generation Sequencing of Clinically Relevant Gene Fusions in Non-Small Cell Lung Cancer
Source: Cancers (Basel). 2021 Jan 4;13(1):139. doi: 10.3390/cancers13010139 (PMC7796105; doi:10.3390/cancers13010139)
Supplement: Supplementary file 1 [file cancers-13-00139-s001.zip › Supplementary files/Supplementary Table 6.docx]

**Supplementary Table 6.** Results obtained on FN – ETOH sample.

| Locus | Type | Filter | Genes  (exons) | Read counts | Detection |
| --- | --- | --- | --- | --- | --- |
| chr6:170871321 | EXPR_CON^-^ | PASS | *TBP* | 64528 | Present |
| chr12:53585787 | EXPR_CON^-^ | PASS | *ITGB7* | 4321 | Present |
| chr8:128751265 | EXPR_CON^-^ | PASS | *MYC* | 107905 | Present |
| chr11:118960975 | EXPR_CON^-^ | PASS | *HMBS* | 66435 | Present |
| chr1:156104319 | EXPR_CON^-^ | PASS | *LMNA* | 130763 | Present |

Abbreviations: chr: chromosome; *EML4*: Echinoderm Microtubule-Associated Protein-Like 4; ETOH: ethanol; FN: fusion negative; *HMBS*: Hydroxymethylbilane Synthase; *ITGB7*: Integrin Subunit Beta 7; *LMNA*: Lamin A/C; *TBP*: TATA-Box Binding Protein; *MYC*: MYC Proto-Oncogene, BHLH Transcription Factor.
