# Supplementary material for: RNA-Based Assay for Next-Generation Sequencing of Clinically Relevant Gene Fusions in Non-Small Cell Lung Cancer
Source: Cancers (Basel). 2021 Jan 4;13(1):139. doi: 10.3390/cancers13010139 (PMC7796105; doi:10.3390/cancers13010139)
Supplement: Supplementary file 1 [file cancers-13-00139-s001.zip › Supplementary files/Supplementary Table 7.docx]

**Supplementary Table 7.** Fusion positive results obtained on FP – MEOH residual RNA sample after one month of storage at -20°C.

|  | *ALK* | *ROS1* ex 32 | *ROS1* ex34 | *ROS1* ex 35-36 | *RET* ex12 | *RET* ex 8-11 | *MET* ex 14 skipping | *NTRK1* ex 9-10 | *NTRK1* ex 10 | *NTRK1* ex 11-12del | *NTRK1* ex 12 | *NTRK2* ex 12-15 | *NTRK2* ex 16-17 | *NTRK3* ex 14 | *NTRK3* ex 15 |
| --- | --- | --- | --- | --- | --- | --- | --- | --- | --- | --- | --- | --- | --- | --- | --- |
| FP - MEOH | MUT | MUT | MUT | WT | MUT | WT | WT | WT | WT | WT | WT | WT | WT | WT | MUT |

Abbreviations: *ALK*: Anaplastic Lymphoma Kinase; ex: exon; FP: fusion positive; MEOH: methanol; *MET*: MET Proto-Oncogene, Receptor Tyrosine Kinase; *NTRK*: Neurotrophic Receptor Tyrosine Kinase; *RET*: Rearranged During Transfection; *ROS1*: ROS Proto-Oncogene 1, Receptor Tyrosine Kinase.
