# Supplementary figures and images for: RNA-Based Assay for Next-Generation Sequencing of Clinically Relevant Gene Fusions in Non-Small Cell Lung Cancer
Source: Cancers (Basel). 2021 Jan 4;13(1):139. doi: 10.3390/cancers13010139 (PMC7796105; doi:10.3390/cancers13010139)

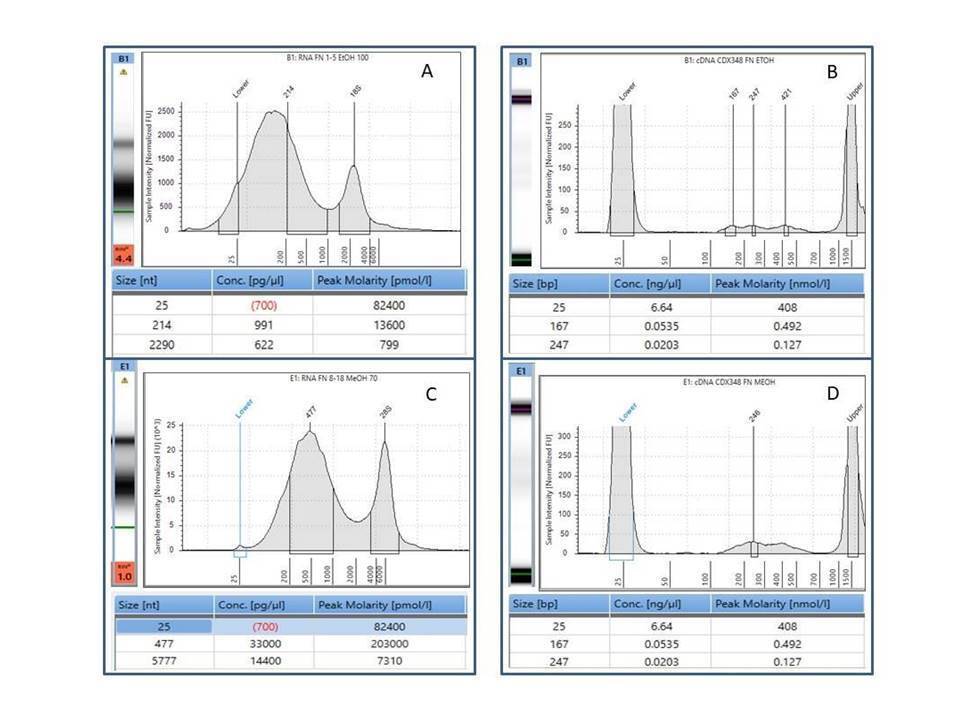

Supplement: Supplementary file 1 [file cancers-13-00139-s001.zip › Supplementary files/Supplementary Figure 1.jpg]

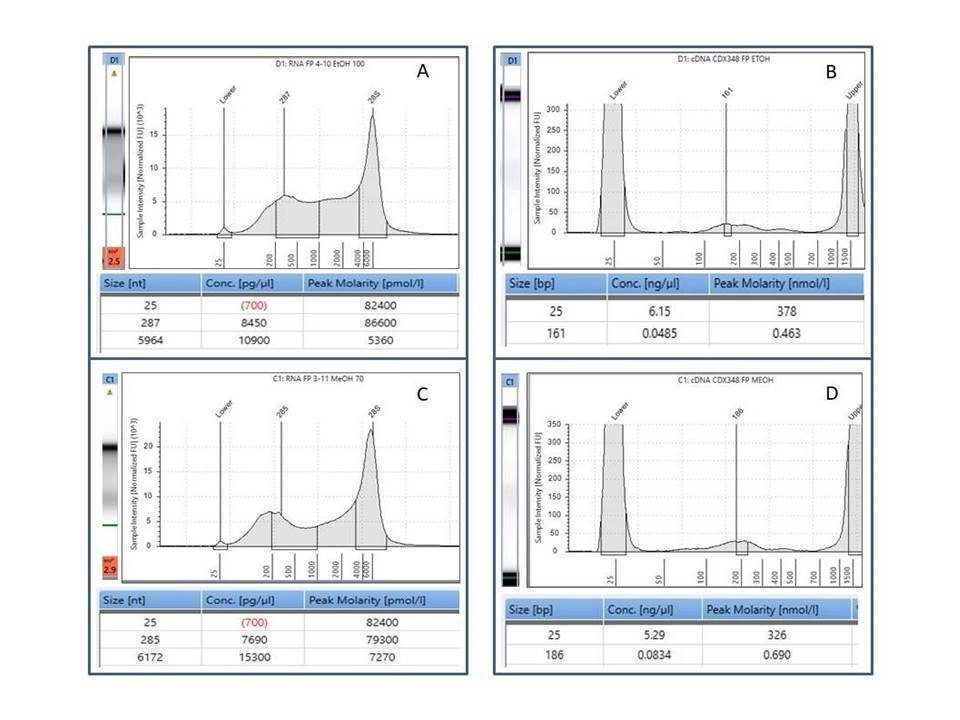

Supplement: Supplementary file 1 [file cancers-13-00139-s001.zip › Supplementary files/Supplementary Figure 2.jpg]
